# Genome-wide association study identifies candidate genes and favorable haplotypes for maize lodging-related traits

**DOI:** 10.1186/s12870-026-09725-x

**Published:** 2026-08-14

**Authors:** Huafeng Liu, Zihan Lu, Feiyu Ye, Dongling Zhang, Chenchen Ma, Yinghui Song, Yanyu Tian, Kaihang Yao, Shulei Guo, Guo Li, Desheng Hu, Xin Zhang, Jihua Tang, Liru Cao, Xiaomin Lu

**Affiliations:** 1 Henan Academy of Agricultural Sciences, Henan Agricultural University, Zhengzhou, China; 2 The Shennong Laboratory, Zhengzhou, China; 3 State Key Laboratory of Maize Bio-Breeding, Zhengzhou, China

**Keywords:** Maize, Lodging resistance, Genome-wide association study, Favorable haplotypes, *ZmGUX1*

## Abstract

**Background:**

Lodging is a major constraint limiting maize (*Zea mays* L.) productivity and yield stability. Both stalk mechanical strength and root architecture contribute to lodging resistance. However, their combined genetic basis remains incompletely understood. This study jointly evaluated stalk mechanical and root architectural traits within a unified genome-wide association study framework to provide a more integrated understanding of the genetic architecture underlying maize lodging resistance.

**Results:**

A panel of 300 maize inbred lines were evaluated for ten lodging-related traits across three environments. Genome-wide association studies based on 1,213,985 high-quality SNPs identified 1,175 significant trait-associated SNPs, of which 458 were consistently detected by at least two statistical models. Candidate gene analysis identified 26 genes potentially involved in lodging-related traits, including genes associated with cell wall organization, carbohydrate metabolism, polysaccharide biosynthesis, and glycosyltransferase activity. Haplotype analysis revealed significant phenotypic differences among haplotypes of 20 candidate genes and identified multiple favorable haplotypes associated with enhanced stalk mechanical strength and root architecture characteristics. Evaluation of 36 representative maize inbred lines revealed substantial variation in the accumulation of favorable alleles and suggested that tropical germplasm may provide useful allelic variation for lodging-resistance improvement. Among the candidate genes, *ZmGUX1* (*Zm00001d038229*) was associated with total nodal root number. A *Ds* element insertion mutant of *ZmGUX1* exhibited a significant reduction in nodal root number and altered root tissue organization, supporting a role for *ZmGUX1* in nodal root development.

**Conclusions:**

These findings provide an integrated view of the genetic basis of lodging-related stalk and root traits in maize. The identified loci, candidate genes, and favorable haplotypes provide promising genetic resources for further functional validation and may facilitate marker-assisted selection and genomic breeding for improved lodging resistance.

**Supplementary Information:**

The online version contains supplementary material available at https://doi.org/10.1186/s12870-026-09725-x.

## Introduction

Lodging is one of the major constraints limiting maize production and yield stability. It disrupts the normal spatial arrangement of plants, interferes with water and nutrient transport, reduces harvesting efficiency, and ultimately compromises crop yield and quality. In cereal crops such as maize, lodging is generally classified into two major types: stalk lodging and root lodging. Stalk lodging is characterized by bending or breakage of the stalk near the ground surface, whereas root lodging occurs when the root system fails to provide sufficient anchorage, resulting in plant displacement or even complete toppling [1, 2]. Lodging is caused by the combined effects of genetic background, agronomic management practices, planting density, and diverse environmental factors, including both biotic and abiotic stresses [3, 4]. Therefore, elucidating the genetic basis of lodging resistance is essential for improving maize structural stability and provides an important theoretical basis for molecular breeding of lodging-resistant cultivars.

Stalk lodging is closely associated with stem mechanical strength, anatomical structure, and stem tissue composition. The contents and proportions of cellulose, hemicellulose, and lignin directly affect stalk-related morphological and mechanical traits, including stalk bending strength (SBS), rind penetrometer resistance (RPR), and stalk crushing strength (SCS) [5–8]. These traits are significantly correlated with stalk strength and stalk lodging resistance under field conditions [9–11]. Several studies have identified QTLs and SNPs associated with stalk-strength-related traits [8, 12, 13]. For example, 94 QTLs associated with stalk strength were detected using genome-wide association studies (GWAS) [8]. RPR at different maize internodes was associated with 17, 14, and 17 QTLs, respectively, each explaining 3.83%–17.25% of phenotypic variation [12]. A multi-locus GWAS conducted in 257 inbred lines identified 29, 34, and 48 stable loci associated with stalk diameter (SD), SBS, and RPR, respectively [14], while 14, 14, and 16 QTLs for SD, SBS, and RPR were identified by single-environment mapping in an IBM Syn10 DH population [15]. At the molecular level, miR528 has been shown to affect lodging resistance under nitrogen-rich conditions by regulating lignin biosynthesis [16]. The major QTL *stiff1* was cloned by combining map-based cloning and association mapping, and its positive effect on stalk strength was attributed to increased cellulose and lignin accumulation in the cell wall [17]. Transcriptomic and metabolomic comparisons between lodging-resistant and lodging-susceptible genotypes further identified differentially expressed genes (DEGs) involved in lignin metabolism and polysaccharide biosynthesis [11, 18]. In addition, 29 significant SNPs explaining 7.19%–15.03% of phenotypic variation were identified for RPR, SD, stalk length (SL), and dry matter content, among which *Zm00001d014449* was repeatedly detected and accounted for 9.95%–10.84% of phenotypic variation. This gene is mainly expressed in tissues with active cell division and differentiation and may participate in vascular bundle development and cell wall biosynthesis [13]. These studies indicate that stalk strength is controlled by complex genetic and molecular regulatory networks.

Root system architecture (RSA) also plays a fundamental role in determining plant anchorage and root lodging resistance in maize. As the primary interface between plants and soil, the root system not only facilitates water and nutrient acquisition but also provides the mechanical support required to maintain plant stability. Variations in RSA, including root number (RN), root length (RL), root diameter (RDI), root angle (RA), root depth (RD), root volume (RV), and spatial distribution, can substantially influence anchorage strength and lodging resistance. Previous studies have shown that optimized root architectural traits can improve both resource acquisition efficiency and adaptation to environmental stresses [19–21]. Among different maize root types, nodal roots, including crown roots and brace roots, are major structural components contributing to plant anchorage. Brace roots emerge from aboveground stem nodes and can penetrate the soil to form additional support systems, thereby reinforcing root-soil interactions and improving plant stability. Comparative genetic studies revealed that modern maize generally possesses more aerial nodal roots and fewer crown roots than its wild relatives, a root configuration that may enhance root lodging resistance and improve deep-water and nitrogen acquisition under dense planting conditions [22]. Biomechanical analyses further demonstrated that brace roots contribute substantially to anchorage strength, and root lodging resistance is associated with multiple brace-root-related traits, including the number of brace root (NBR) whorls entering the soil, RDI, RA, and root spread width (RSW) [23]. These findings suggest that root lodging resistance is determined by the coordinated effects of multiple root architectural components rather than by a single root trait.

Recent quantitative genetic and molecular studies have further revealed the genetic basis of root architectural variation associated with lodging resistance. QTL mapping and GWAS have identified loci controlling the total nodal root number (TNR), RA, RD, root tier number (RTN), and RN [22, 24]. In a diverse maize association panel, brace root number, tier number, and penetration resistance exhibited relatively high heritability and were positively associated with yield-related traits, indicating that improvement of root architecture may simultaneously enhance lodging resistance and agronomic performance [24]. Several candidate genes involved in hormone biosynthesis, signal transduction, oxidative metabolism, and cell wall development have also been proposed to regulate brace root formation and root mechanical properties. Auxin, a central regulator of root initiation, elongation, gravitropic responses, and lateral root formation, has been shown to participate in root-mediated lodging resistance. *ZmYUC2* and *ZmYUC4*, two genes involved in local auxin biosynthesis, regulate brace root gravitropism and root angle formation. Mutations in these genes lead to larger brace root angles (RA), increased soil contact area, and enhanced lodging resistance under high-density planting conditions [25]. Similarly, natural variation in *ZmRLR1* affects lateral root initiation and elongation by regulating auxin and ascorbate homeostasis, resulting in increased total RL, RV, and anchorage strength [26]. Previous studies have suggested that LBD transcription factors are involved in maize root development. In particular, *ZmLBD12* has been identified as a root-specific LBD gene that promotes lateral root formation, making it a promising candidate for improving root architecture and lodging resistance in maize [27]. Recent studies have shown that some genes coordinately regulate both root system architecture and stalk-related traits. For example, *ZmCCT* (*stiff2*) has been reported to simultaneously regulate root system architecture and stalk bending strength, thereby enhancing resistance to both root lodging and stalk lodging [28].

Although considerable progress has been made in identifying stalk- and root-related traits associated with lodging resistance, the genetic architecture of lodging resistance remains incompletely understood. Lodging resistance is a complex quantitative trait involving multiple organs, developmental processes, and regulatory pathways, and the loci controlling lodging-related traits across diverse maize germplasm remain insufficiently characterized. Moreover, previous genome-wide association studies have generally investigated stalk mechanical traits or root architectural traits separately, whereas the genetic relationship between these complementary components of lodging resistance has received much less attention. Therefore, integrating both trait categories within a unified GWAS framework may provide a more comprehensive understanding of the genetic architecture underlying maize lodging resistance. In this study, a diverse maize association panel consisting of 300 inbred lines was evaluated for ten lodging-related traits across three environments. Genome-wide association studies were performed using 1,213,985 high-quality SNPs to identify loci associated with stalk mechanical and root architectural traits. Candidate genes were further investigated through expression profiling and haplotype analysis, and favorable haplotypes were surveyed in representative maize germplasm. In addition, the function of *ZmGUX1*, a candidate gene associated with TNR, was characterized using a *Ds* insertion mutant. By jointly dissecting the genetic basis of stalk mechanical and root architectural traits, this study provides a more integrated understanding of the genetic architecture underlying maize lodging resistance and valuable genetic resources for molecular breeding.

## Materials and methods

### Plant materials and experimental design

A diverse association panel comprising 300 maize (*Zea mays* L.) inbred lines was grown in three environments in China during 2024, including Zhangye (ZY; 38°56′ N, 100°27′ E, Gansu Province), Zhengzhou (ZZ; 34°46′ N, 113°41′ E, Henan Province), and Sanya (SY; 18°16′ N, 109°30′ E, Hainan Province). The experiments at each location followed a randomized complete block design with two replications. Each accession was planted in a single-row plot measuring 3 m in length, with 60 cm between rows and 20 cm between plants, resulting in a planting density of approximately 83,333 plants ha⁻^1^. Field management practices, including irrigation, fertilization, and pest control, were performed according to local recommendations.

### Phenotypic evaluation

Phenotypic assessments were performed 20 days after pollination for a set of lodging-related traits. Root architectural traits included root angle (RA), root spread width (RSW), root depth (RD), total nodal roots number (TNR), and root dry weight (RDW). Stalk mechanical traits comprised stalk bending strength at 30° measured at 20 cm above ground (SBS30-20), 40 cm above ground (SBS30-40), and ear height (SBS30-Ear), together with rind penetrometer resistance (RPR) and stalk crushing strength (SCS). All mechanical measurements were obtained using a YYD-1 plant stem strength tester (Zhejiang Taipu Yunnong Technology Co., Ltd., Hangzhou, China) equipped with corresponding sensors. Ten plants were randomly sampled from each subplot, and trait values were averaged across plants prior to subsequent phenotypic and genome-wide association analyses.

### Phenotypic data processing and statistical analysis

Phenotypic data of the 10 lodging-related traits (stalk traits: stalk bending strength at 30° [SBS30-20, SBS30-40, SBS30-Ear], RPR, and SCS; root traits: RA, RSW, RD, TNR, and RDW) were analyzed using a linear mixed model cross the three environments. The model was specified as:

$${y}_{ijk}=\mu +{G}_{i}+{E}_{j}+(GE{)}_{ij}+{B}_{k}({E}_{j})+{\varepsilon}_{ijk}$$

where ${y}_{ijk}$ is the observed phenotypic value of genotype *i* in environment *j* and block *k*, *μ* is the overall mean, ${G}_{i}$ is the effect of the *i* th genotype, ${E}_{j}$ is the effect of the *j* th environment (location), $(GE{)}_{ij}$ is the genotype-by-environment interaction, ${B}_{k}({E}_{j})$ is the effect of the *k* th block (subplot) within the *j* th environment, and ${\varepsilon}_{ijk}$ is the residual error.

For variance component estimation, genotype, environment, genotype-by-environment interaction, and block-within-environment effects were treated as random effects. Analysis of variance (ANOVA) was performed to evaluate the significance of genotype, environment, and genotype-by-environment interaction effects for each trait. Variance components were estimated using the lme4 package in R, and the significance of random effects was assessed using likelihood ratio tests implemented in the lmerTest package. Broad-sense heritability across environments was calculated on an entry-mean basis as:

$${H}^{2}=\frac{{r}_{g}^{2}}{{r}_{g}^{2}+{r}_{ge}^{2}/e+{r}_{e}^{2}/(re)}$$

where ${\sigma}_{G}^{2}$ is the genotypic variance, ${\sigma}_{GE}^{2}$ is the genotype-by-environment interaction variance, ${\sigma}_{\varepsilon }^{2}$ is the residual variance, *e* is the number of environments, and *r* is the number of replications per environment.

Best linear unbiased predictions (BLUP) values for each genotype were estimated from the above mixed model to obtain genotype performance adjusted for environmental and block effects. In the BLUP model, genotype was treated as a random effect so that predicted genotypic values could be extracted for all accessions across environments. These BLUP values were used as the primary phenotypic inputs for GWAS and for pairwise trait correlation analysis. In addition, single-environment phenotypic data were used for descriptive comparisons among environments. Descriptive statistics, including mean, standard deviation, coefficient of variation (CV), and range, were calculated for each trait in each environment and for BLUP values across environments. Pairwise Pearson correlation coefficients among the 10 traits were calculated based on BLUP values to evaluate the relationships between stalk mechanical traits and root architectural traits while reducing environmental noise. All statistical analyses were conducted in R (v4.5).

### Genotypic analysis and genome-wide association study

The association panel consisted of 300 maize inbred lines whose genotype data were obtained through whole-genome resequencing. Raw SNPs were filtered by removing markers with a missing rate > 0.01 or a minor allele frequency (MAF) < 0.05, resulting in 1,213,985 high-quality SNPs for subsequent analyses. Population structure was evaluated by principal component analysis (PCA), and the first five principal components were included as fixed-effect covariates in genome-wide association analysis. ADMIXTURE analysis (K = 6) was performed to infer subgroup membership, and pairwise kinship coefficients were estimated using the centered identity-by-state method.

GWAS was conducted using the BLUP values estimated across all environments. The BLUP values were used to minimize environmental variation and improve the accuracy of genetic effect estimation. GWAS was performed using the GLM, MLM, and FarmCPU models implemented in rMVP [29]. The MLM incorporated both population structure and kinship matrices to control for false-positive associations. Significant marker-trait associations were identified using a modified Bonferroni threshold (*P* < 1/N). The phenotypic variance explained (PVE) by each significant SNP was estimated using the EMMA algorithm based on REML-derived variance components and expressed as the percentage of total phenotypic variance attributable to the marker effect.

Significant SNPs were annotated using SnpEff (v5.4) based on the maize B73 reference genome (RefGen_v4). Candidate genes were identified when significant SNPs were located within gene bodies or within the 2-kb upstream promoter region and 2-kb downstream region of annotated genes. Gene functional information was obtained from the MaizeGDB (https://www.maizegdb.org) and Ensembl Plants databases (https://plants.ensembl.org).

### Candidate gene expression analysis

To further evaluate the biological relevance of the identified candidate genes, tissue-specific expression profiles were analyzed using publicly available RNA-seq datasets from the qTeller database (https://qteller.maizegdb.org/). Expression data from diverse maize tissues, including roots, stems, leaves, tassels, ears, and kernels, were retrieved based on the B73 reference genome annotation. The normalized expression values provided by qTeller were used to compare expression abundance among tissues, and bar plots were generated to illustrate tissue-specific expression patterns.

### Candidate gene haplotype analysis and favorable haplotype survey

Candidate gene haplotype analysis was performed using polymorphic SNPs located within each candidate gene and its 2-kb upstream promoter region. Accessions sharing identical allelic combinations were assigned to the same haplotype. Haplotypes represented by fewer than five accessions were excluded from subsequent analyses. Phenotypic differences among haplotypes were evaluated using the Wilcoxon rank-sum test (*P* < 0.05) to assess the effects of natural allelic variation on lodging-related traits.

For genes showing significant haplotype effects, the haplotype associated with superior phenotypic performance was defined as the favorable haplotype. The distribution of favorable haplotypes among maize subpopulations was subsequently investigated using the resequencing dataset. To evaluate their breeding utilization, 36 representative maize inbred lines, including 16 widely used Chinese elite inbred lines and 20 tropical CIMMYT lines, were selected for favorable haplotype survey. Genotypic information of the corresponding polymorphic sites was extracted, and the presence or absence of favorable haplotypes was determined for each line. The number of favorable alleles carried by each inbred line was calculated by summing favorable haplotypes across all candidate genes. The distribution and frequency of favorable haplotypes among germplasm groups and representative breeding materials were visualized using heatmaps generated in R.

### Mutant identification and phenotypic analysis

A *Ds* element insertion mutant of *ZmGUX1* (*Zm00001d038229*) was obtained from the maize *Ac/Ds* transposon mutant library [30]. The insertion site was verified by PCR amplification and sequencing, primer sequences: ZmGUX1-F: GCCAGTGGCCATCTTGAGAGTTG, *Ds*3: AACGGTAGAGGTATTTTACCGACC. Wild-type (WT) and homozygous *zmgux1* mutant plants were grown under the same conditions. At the seedling stage (V3-stage), RL and TNR were recorded. RL was measured from the root-shoot junction to the root tip, and TNR was determined by counting all emerged nodal roots per plant. At least 15 plants were evaluated for each genotype.

Mature nodal roots were collected from V6-stage WT and *zmgux1* seedlings and fixed in FAA solution (50% ethanol: formaldehyde: acetic acid, 18:1:1, v/v/v). Samples were dehydrated through a graded ethanol series, embedded in paraffin, and sectioned using a rotary microtome. Transverse sections were stained with 0.05% toluidine blue O and observed under a light microscope.

## Results

### Phenotypic variation of lodging-related traits

**Fig. 1:**
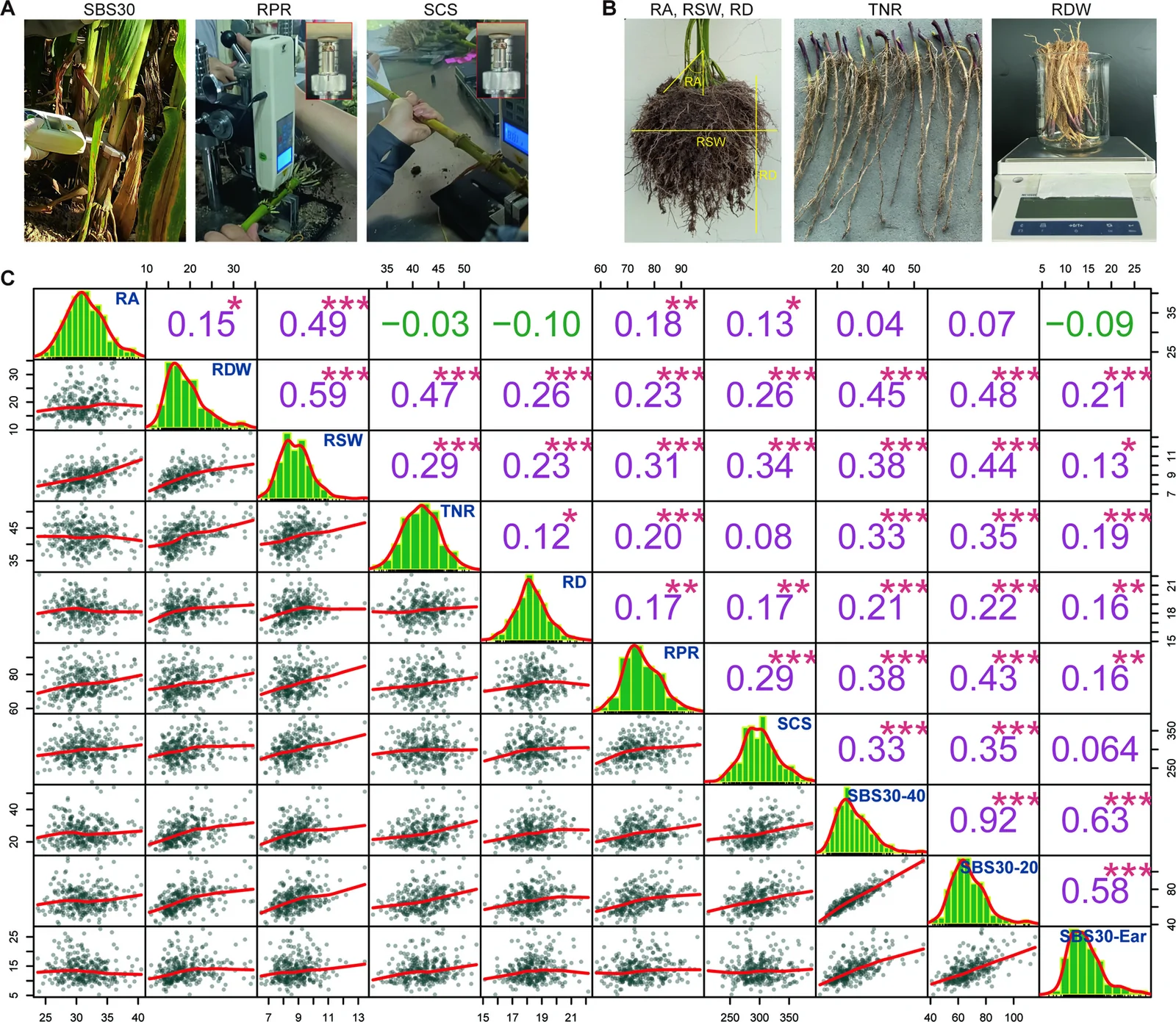
Phenotyping and correlation analysis of lodging-related traits. **A** Phenotyping methods for stalk lodging-related traits.** B** Phenotyping methods for root lodging-related traits. **C** Phenotypic correlations among all measured traits in 300 inbred lines. RA, root angle; RDW, root dry weight; RSW, root spread width; TNR, total nodal root number; RD, root depth; RPR, rind penetrometer resistance; SCS, stalk crushing strength; SBS30-20, SBS30-40 and SBS30-Ear, stalk bending strength measured at 30° at 20 cm above ground, 40 cm above ground and ear height, respectively. Significance levels: *P < 0.05, **P < 0.01, ***P < 0.001

Ten lodging-related traits, including five stalk mechanical traits (SBS30-20, SBS30-40, SBS30-Ear, RPR, and SCS) and five root architectural traits (RA, RDW, RSW, TNR, and RD), were evaluated across three environments (Fig. 1A–B; Table 1). All traits exhibited substantial phenotypic variation, with coefficients of variation ranging from 13.8% to 51.9%. Broad-sense heritability (*H*^2^) ranged from 72.2% to 94.7%, indicating that genetic factors contributed substantially to phenotypic variation. Analysis of variance revealed significant effects of genotype, environment, and genotype-by-environment interaction for all traits (Table 1). Therefore, BLUP values were calculated and used for subsequent analyses. Based on BLUP values, all traits showed continuous variation and approximately normal distributions, consistent with quantitative inheritance (Fig. 1C). Correlation analysis revealed strong positive relationships among stalk mechanical traits and among root architectural traits, while several root traits were also significantly correlated with stalk-related traits, suggesting coordinated contributions of root architecture and stalk strength to lodging resistance.

**Table 1:** Descriptive statistics and ANOVA for lodging-related traits

Trait	Location	Min	Max	Mean_SD	CV(%)	Heritability(%)	E	G	GxE
RPR	ZA	10	126.2	42.1 ± 21.9	51.9	82.1	1060.9^***^	22.7^***^	4.1^***^
	ZB	9.6	180.3	69.4 ± 29.5	42.5				
	ZC	17	142.2	55.8 ± 20.7	37.1				
	Blup	28.7	126.1	62.6 ± 18	28.7				
SCS	ZA	74.4	564.9	294.9 ± 69.5	23.6	88.3	7.5^***^	14.7^***^	1.7^***^
	ZB	112	495.2	287.2 ± 66.3	23.1				
	ZC	140	482.4	291.0 ± 56.6	19.5				
	Blup	158.8	447.7	289.1 ± 49	16.9				
SBS30_20	ZA	6.5	145.9	46.3 ± 19.2	41.5	80.8	205.8^***^	6.9^***^	1.3^***^
	ZB	6	194.7	59.8 ± 26.7	44.7				
	ZC	7.4	180.9	65.1 ± 25.3	38.9				
	Blup	31.3	136.7	62.4 ± 14.9	23.8				
SBS30_40	ZA	3.7	62.6	24.5 ± 10.6	43.2	85.7	26.0^***^	11.9^***^	1.7^***^
	ZB	5	109.2	26.7 ± 12.1	45.3				
	ZC	2	85.4	24.2 ± 11.7	48.3				
	Blup	10.9	59.6	25.5 ± 8.3	32.4				
SBS30_Ear	ZA	0.7	35.9	10.5 ± 5.0	47.5	94.7	13.8^***^	84^***^	4.5^***^
	ZB	0.7	33.9	10.2 ± 4.8	46.9				
	ZC	0.7	35.9	10.2 ± 5.0	48.5				
	Blup	0.9	29.6	10.2 ± 4.5	43.8				
RA	ZA	10.3	62.2	33.6 ± 9.4	27.8	89.3	344.5^***^	16.4^***^	1.8^***^
	ZB	10.9	59.7	27.5 ± 7.9	28.8				
	ZC	13.1	62.2	28.7 ± 7.7	27				
	Blup	14.9	55.5	28.1 ± 6.5	23.2				
RD	ZA	10	31.5	21.2 ± 4.1	19.2	72.2	576.4^***^	11.2^***^	3.1^***^
	ZB	3.6	31	18.1 ± 4.7	26.2				
	ZC	8	31	16.8 ± 3.9	23.3				
	Blup	11.3	24.4	17.4 ± 2.6	15.1				
RDW	ZA	6.5	58.7	20.7 ± 9.2	44.2	91.8	665^***^	52.3^***^	4.3^***^
	ZB	3.9	58.7	15.7 ± 7.3	46.7				
	ZC	3.2	52	16.5 ± 8.4	50.5				
	Blup	5.2	46	16.1 ± 7.2	44.6				
RSW	ZA	4	14.5	8.5 ± 1.7	20.2	80.1	13.7^***^	12.8^***^	2.6^***^
	ZB	4	15	8.8 ± 1.9	21.8				
	ZC	4.5	14.5	8.7 ± 1.6	18.9				
	Blup	5.5	12.6	8.8 ± 1.2	13.8				
TNR	ZA	17	77	41.8 ± 8.8	21.1	86.6	165.7^***^	16.1^***^	2.2^***^
	ZB	18	76	43.3 ± 9.6	22.1				
	ZC	21	83	46.9 ± 11.0	23.5				
	Blup	23.3	69.5	45.1 ± 7.4	16.5				

*** indicates significance at *P* < 0.001

### Genetic diversity and population structure

A total of 183.94 million raw SNPs were obtained from resequencing of 300 maize inbred lines. After filtering SNPs with MAF < 0.05 and missing rate > 0.01, 1,213,985 high-quality SNPs were retained for subsequent analyses (Figure S1–S2). These SNPs were distributed across all ten maize chromosomes, although marker density varied among genomic regions (Figure S3).

**Fig. 2:**
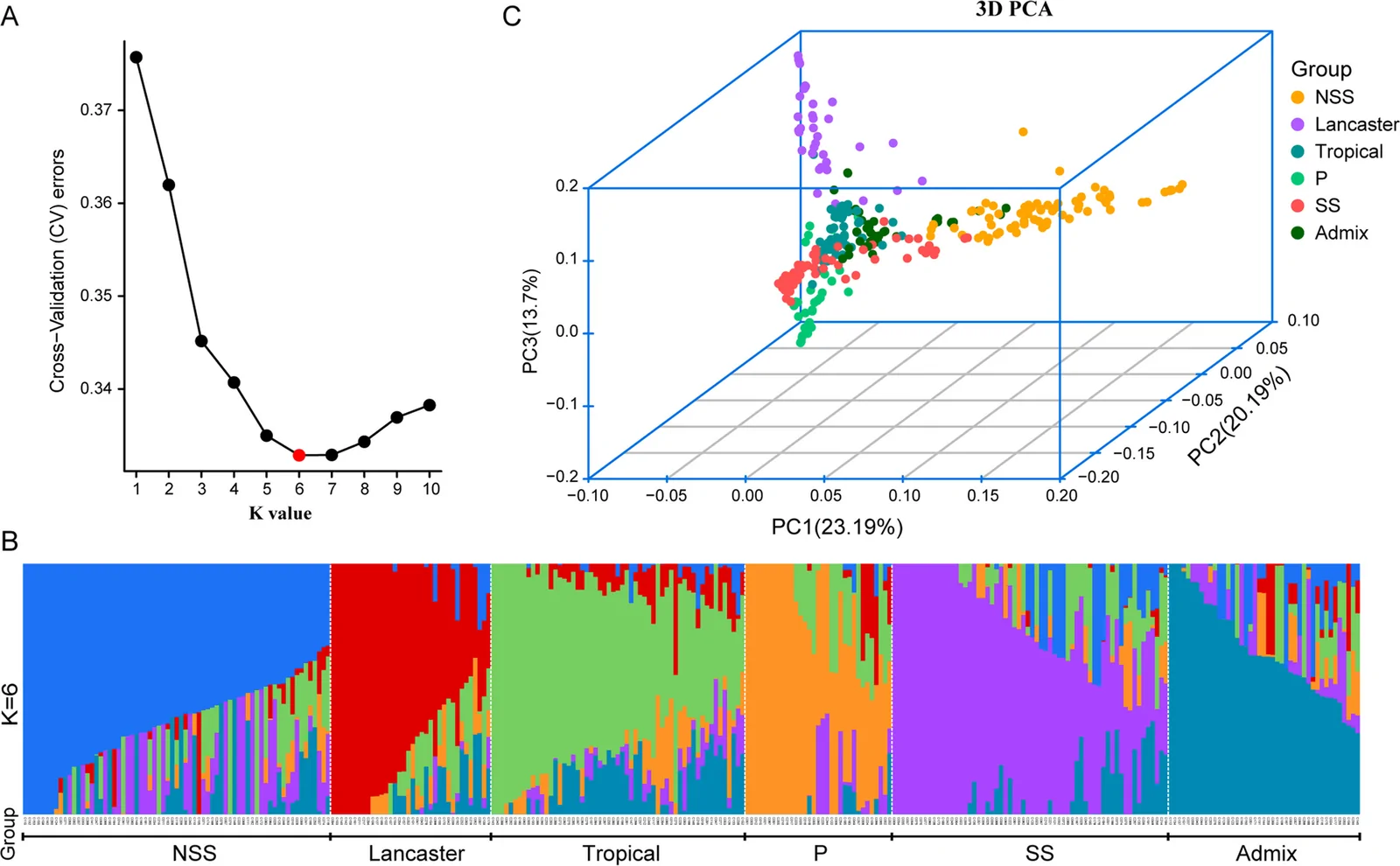
Population structure analysis of the maize association panel. **A** Cross-validation (CV) error estimated by ADMIXTURE under different K values, with the minimum CV error observed at K = 6.** B** Population structure inferred by ADMIXTURE at K = 6, classifying the 300 maize inbred lines into six subgroups: NSS, Lancaster, Tropical, P, SS, and Admix. Each vertical bar represents one inbred line, and different colors indicate the membership proportion of each genetic subgroup.** C** Three-dimensional principal component analysis (3D PCA) of the association panel based on genome-wide SNP markers. The first three principal components explained 23.19%, 20.19%, and 13.70% of the total genetic variation, respectively

Population structure analysis using ADMIXTURE identified K = 6 as the optimal number of subpopulations (Fig. 2A). Accordingly, the association panel was divided into six groups, including NSS, Lancaster, Tropical, P, SS, and Admix (Fig. 2B). PCA generated a clustering pattern largely consistent with the ADMIXTURE results, with the first three principal components explaining 23.19%, 20.19%, and 13.70% of the total genetic variation, respectively (Fig. 2C). These results revealed clear population stratification within the association panel and provided a basis for controlling population structure in subsequent GWAS analyses.

### GWAS identifies loci associated with lodging-related traits

**Fig. 3:**
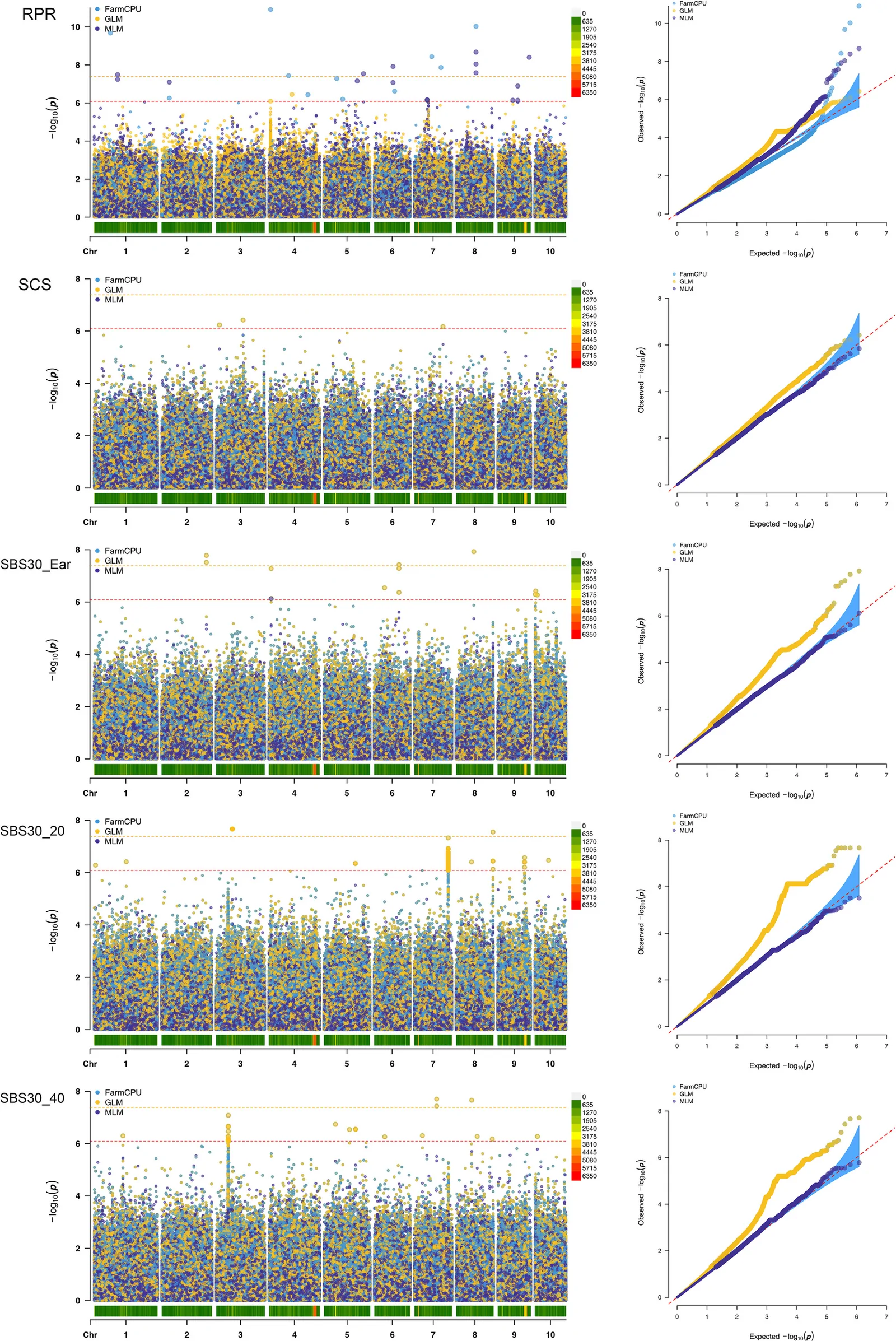
Genome-wide association analysis of stalk lodging-related traits. Manhattan plots and corresponding quantile–quantile (QQ) plots for stalk lodging-related traits, including RPR, SCS, SBS30_20, SBS30_40, and SBS30_Ear. The x-axis represents the genomic positions of SNPs across the 10 maize chromosomes, and the y-axis represents the negative logarithm of the association *p*-values [− log_10_(*p*)]. The GWAS results obtained using the FarmCPU, GLM, and MLM models are represented by light blue, yellow, and purple, respectively. The upper and lower dashed lines indicate the genome-wide significance threshold (0.05/N) and suggestive significance threshold (1/N), respectively, where N represents the total number of SNPs used for association analysis. SNPs exceeding the suggestive significance threshold were considered significantly associated loci and were further used for candidate gene identification

**Fig. 4:**
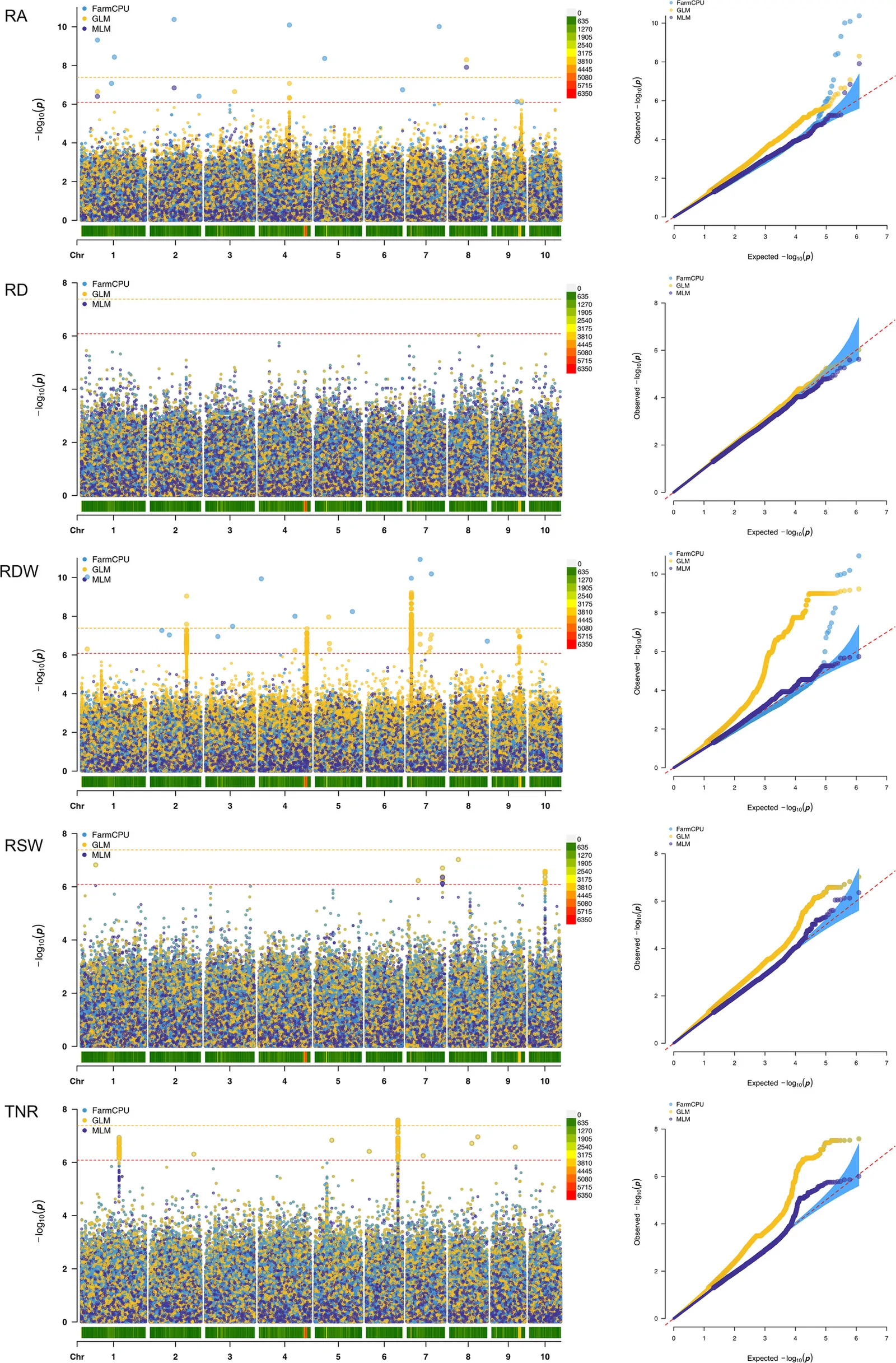
Genome-wide association analysis of root lodging-related traits.Manhattan plots and corresponding QQ plots for root lodging-related traits, including RA, RD, RDW, RSW, and TNR. The x-axis represents the genomic positions of SNPs across the 10 maize chromosomes, and the y-axis represents the negative logarithm of the association p-values [− log_10_(*p*)]. The GWAS results obtained using the FarmCPU, GLM, and MLM models are represented by light blue, yellow, and purple, respectively. The upper and lower dashed lines indicate the genome-wide significance threshold (0.05/N) and suggestive significance threshold (1/N), respectively, where N represents the total number of SNPs used for association analysis. SNPs exceeding the suggestive significance threshold were considered significantly associated loci and were further used for candidate gene identification

GWAS was conducted using 1,213,985 SNPs and three statistical models (GLM, MLM, and FarmCPU) (Figs. 3 and 4). Across the ten lodging-related traits, a total of 1,175 significant SNPs were identified, including 325 associated with stalk-related traits and 850 associated with root-related traits (Table S1). The number of significant associations varied markedly among traits. Among the stalk-related traits, SBS30_20 showed the largest number of significant SNPs, with 251 associations, whereas only three significant SNPs were detected for SCS. Among the root-related traits, RDW had the highest number of significant SNPs, with 689 associations, while no significant SNPs were detected for RD. The number of loci identified also differed among statistical models. For RPR, the MLM detected more significant SNPs than the GLM and FarmCPU, whereas for most of the other traits, the GLM and FarmCPU identified a greater number of associations. Considerable overlap was observed between the GLM and FarmCPU results, with shared significant SNPs detected for SCS, SBS30_20, SBS30_40, SBS30_Ear, RSW, and TNR (Tables S1-S2). Overall, 458 significant SNPs were consistently identified by at least two models, supporting the robustness of these association signals.

Annotation of significant SNPs identified 103 genic variants corresponding to 26 candidate genes (Table 2). Eleven candidate genes were associated with stalk-related traits, whereas fifteen were associated with root-related traits. The phenotypic variation explained by these loci ranged from 5.68% to 11.92%. Several candidate genes encoded proteins related to carbohydrate metabolism and cell wall-associated processes. For example, *Zm00001d027472* encodes a putative callose synthase, *Zm00001d040684* encodes a pectinacetylesterase family protein, and *Zm00001d039231* is predicted to participate in cell wall polysaccharide biosynthesis. Notably, *Zm00001d038229*, a candidate gene associated with TNR, encodes a glycosyltransferase 8 family protein and explained 8.50% of the phenotypic variation. These findings indicate that cell wall polysaccharide metabolism and glycosylation-related processes may be important contributors to genetic variation in maize lodging resistance.

**Table 2:** Candidate genes identified by GWAS for lodging-related traits

Gene	Trait	Model	Chr	Pos	SNP	Mutant type	-log_10_(*P*)	PVE(%)	Description
Zm00001d017308	RPR	MLM	5	192,364,097	5_192364097	intron_variant	7.54	9.79	Transcription factor-related, putative, expressed
Zm00001d036724	RPR	FarmCPU	6	99,056,722	6_99056722	3_prime_UTR_variant	6.62	6.40	TBP-associated factor 5, WD40 repeats
Zm00001d027472	SBS30_20	GLM, FarmCPU	1	6,077,225	1_6077225	intron_variant	6.29	8.09	Callose synthase
Zm00001d021860	SBS30_20	GLM, FarmCPU	7	164,698,437	7_164698437	intron_variant	6.78	8.75	Zinc finger
Zm00001d047464	SBS30_20	GLM, FarmCPU	9	130,960,595	9_130960595	missense_variant	6.21	7.97	Adaptin family protein
Zm00001d040684	SBS30_40	GLM, FarmCPU	3	58,039,695	3_58039695	synonymous_variant	6.65	8.58	Pectinacetylesterase family protein
Zm00001d015818	SBS30_40	GLM, FarmCPU	5	123,624,569	5_123624569	upstream_gene_variant	6.54	8.43	Phospholipase D beta 1
Zm00001d012446	SBS30_40	GLM, FarmCPU	8	174,696,596	8_174696596	premature_start_codon_gain_variant	6.18	7.93	Vacuolar proton ATPase A1
Zm00001d006837	SBS30_Ear	GLM, FarmCPU	2	217,664,540	2_217664540	3_prime_UTR_variant	7.78	10.11	Methionine biosynthesis protein MetW
Zm00001d023311	SBS30_Ear	GLM, FarmCPU	10	2,747,278	10_2747278	missense_variant	6.29	8.09	NB-ARC domain-containing disease resistance protein
Zm00001d023373	SBS30_Ear	GLM, FarmCPU	10	4,260,407	10_4260407	intron_variant	6.42	8.27	small nucleolar RNA-associated SSU e protein 25
Zm00001d007563	RA	FarmCPU	2	234,935,236	2_234935236	missense_variant	6.41	5.68	Retrotransposon protein
Zm00001d039231	RA	FarmCPU	6	173,934,188	6_173934188	splice_region_variant	6.75	6.28	Involved in the synthesis of glucuronoxylan hemicellulose in secondary cell walls
Zm00001d009792	RA	GLM, MLM	8	81,584,094	8_81584094	intron_variant	8.29	10.80	ATP10 protein
Zm00001d047812	RA	GLM, FarmCPU	9	142,391,098	9_142391098	intron_variant	6.16	7.92	OSBP(oxysterol binding protein)-related protein 3C
Zm00001d028225	RDW	GLM, FarmCPU	1	27,264,890	1_27264890	intron_variant	10.03	10.71	Pentatricopeptide repeat (PPR-like) superfamily protein
Zm00001d053375	RDW	GLM	4	228,266,857	4_228266857	intron_variant	7.08	9.16	C2H2 and C2HC zinc fingers superfamily protein
Zm00001d019169	RDW	GLM	7	20,099,761	7_20099761	missense_variant	6.84	8.84	Kinase associated protein phosphatase
Zm00001d019172	RDW	GLM	7	20,302,733	7_20302733	5_prime_UTR_variant	9.13	11.92	Alpha/beta-Hydrolases superfamily protein
Zm00001d019173	RDW	GLM	7	20,343,490	7_20343490	3_prime_UTR_variant	8.99	11.73	Protein of unknown function (DUF630 and DUF632)
Zm00001d019182	RDW	GLM	7	20,583,662	7_20583662	missense_variant	6.27	8.07	Eukaryotic translation initiation factor 3E
Zm00001d019184	RDW	GLM	7	20,586,902	7_20586902	5_prime_UTR_variant	6.42	8.26	KDPG and KHG aldolase
Zm00001d019206	RDW	GLM	7	21,890,512	7_21890512	intron_variant	7.05	9.12	Tryptophan/tyrosine permease
Zm00001d047471	RDW	GLM	9	131,193,230	9_131193230	intron_variant	6.13	7.87	INO80 ortholog, DNA-binding domain
Zm00001d006496	TNR	GLM, FarmCPU	2	209,840,459	2_209840459	3_prime_UTR_variant	6.31	8.11	RNA-binding (RRM/RBD/RNP motifs) family protein
Zm00001d038229	TNR	GLM, FarmCPU	6	152,049,292	6_152049292	stop_gained	6.59	8.50	Belongs to the glycosyltransferase 8 family protein

**Fig. 5:**
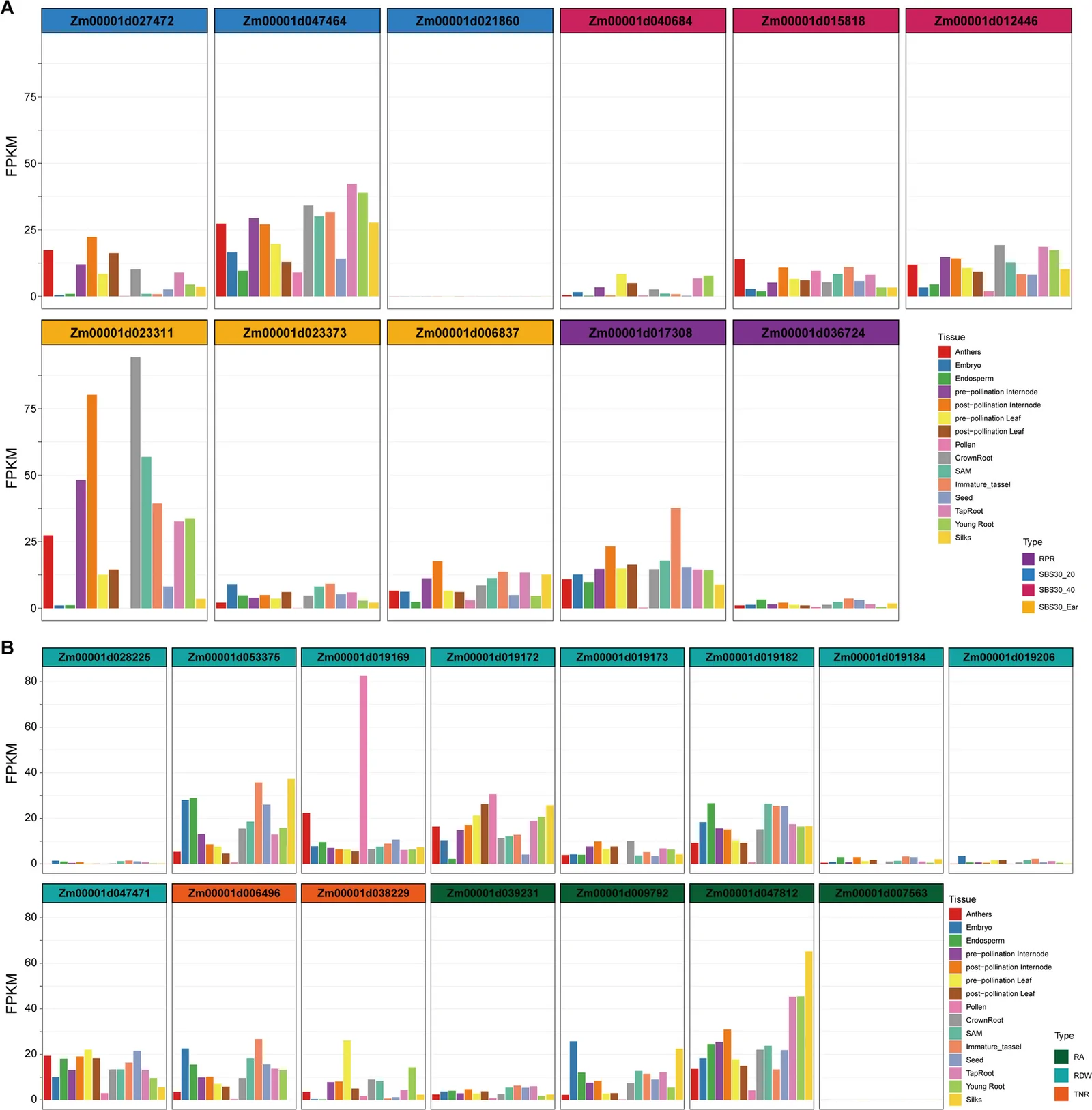
Tissue-specific expression profiles of candidate genes associated with lodging resistance. **A** Expression patterns of candidate genes associated with stalk lodging-related traits (RPR, SBS30_20, SBS30_40, and SBS30_Ear). **B** Expression patterns of candidate genes associated with root lodging-related traits (RA, RDW, and TNR). Gene expression levels across different maize tissues were obtained from publicly available transcriptome datasets. The colored boxes above each panel indicate candidate genes associated with different lodging-related traits, including RPR, SBS30_20, SBS30_40, SBS30_Ear, RA, RDW, and TNR. The colors of the bars represent different maize tissues, as indicated in the Tissue legend

To further evaluate the biological relevance of the identified candidate genes, tissue-specific expression patterns were examined using publicly available transcriptome datasets. Several candidate genes associated with stalk-related traits exhibited relatively high expression levels in stem-associated tissues. For example, *Zm00001d027472*, *Zm00001d047464*, *Zm00001d023311*, and *Zm00001d006837* showed preferential expression in internodes, stems, or other vegetative tissues related to stalk development (Fig. 5A). In contrast, several root-associated candidate genes, including *Zm00001d038229* and *Zm00001d047812*, were predominantly expressed in root tissues. Notably, *Zm00001d038229*, which was associated with TNR, displayed relatively high expression in crown roots and young roots (Fig. 5B). Overall, the tissue-specific expression patterns of these candidate genes were generally consistent with their GWAS trait associations, providing supporting evidence for their potential involvement in the corresponding lodging-related traits.

### Haplotype analysis identifies favorable haplotypes associated with lodging-related traits

**Fig. 6:**
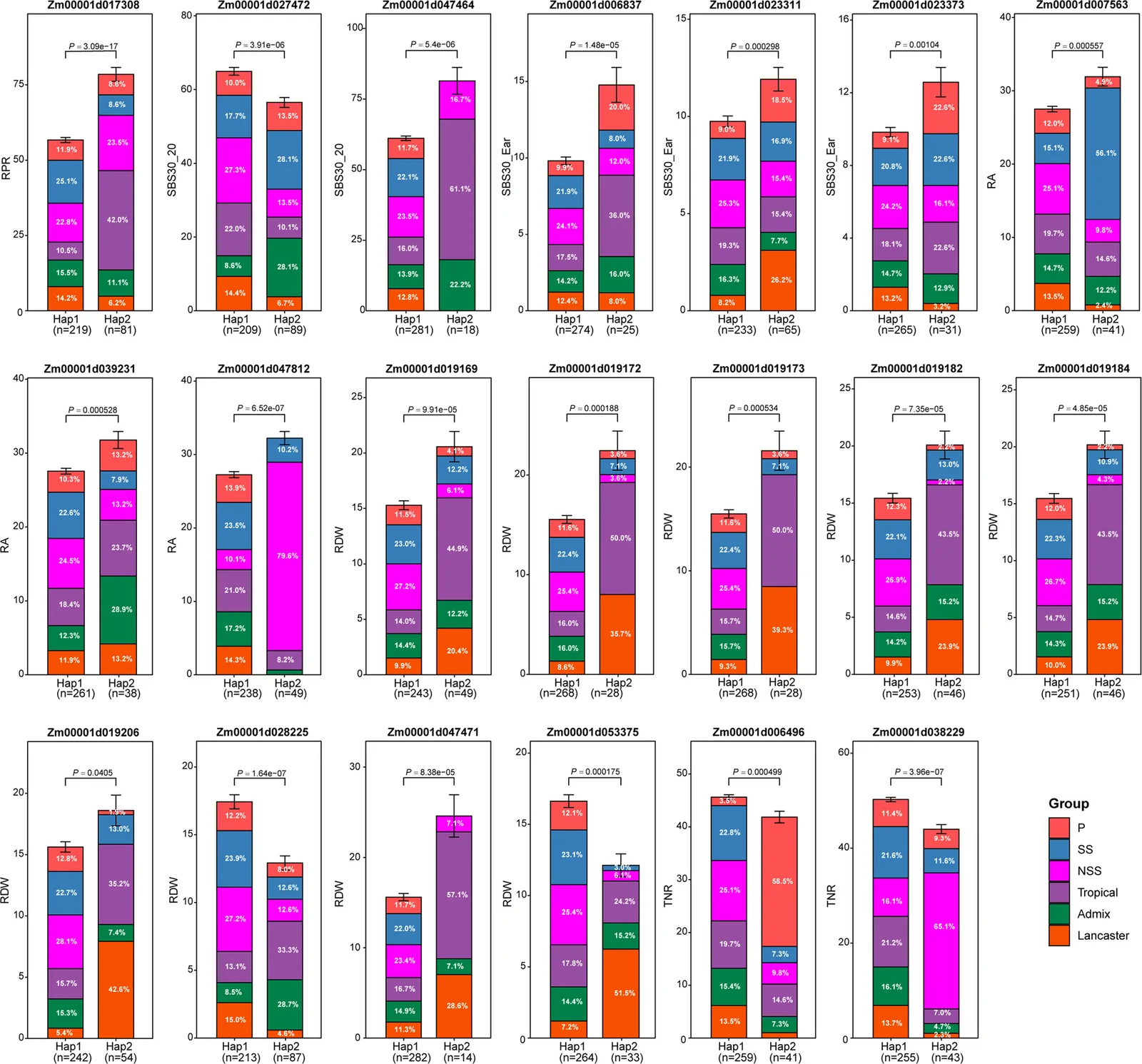
Haplotype analysis of candidate genes associated with lodging-related traits. Phenotypic differences between haplotypes were evaluated for 20 candidate genes identified by GWAS. Bars represent mean values ± SE of the corresponding traits for each haplotype, with sample sizes shown in parentheses. *P* values indicate the significance of phenotypic differences between haplotypes. Stacked bars represent the relative proportions of maize subpopulations within each haplotype. The distribution patterns of favorable haplotypes across germplasm groups were used to assess the genetic diversity and population specificity of superior alleles associated with lodging-related traits

Haplotype analyses were conducted for the 26 candidate genes identified by GWAS. Significant phenotypic differences among haplotypes were detected for 20 genes, indicating associations between natural allelic variation at these loci and lodging-related traits (Fig. 6; Table 3). Favorable haplotypes of *Zm00001d027472*, *Zm00001d040684*, and *Zm00001d047464* were associated with greater stalk bending strength, whereas favorable haplotypes of *Zm00001d039231*, *Zm00001d019184*, and *Zm00001d038229* were associated with higher values of root-related traits.

**Table 3:** Haplotype composition, frequency, and favorable haplotypes of candidate genes associated with lodging-related traits

Traits	Gene	Haplotype	Allele	Frequency	Phenotypic effect
RPR	Zm00001d017308	Hap001	TT	219	
		Hap002	CC	81	Favorable
SBS30_20	Zm00001d027472	Hap001	CC	209	Favorable
		Hap002	TT	89	
	Zm00001d047464	Hap001	CC	281	
		Hap002	TT	18	Favorable
SBS30_Ear	Zm00001d006837	Hap001	CC TT	274	
		Hap002	TT CC	25	
	Zm00001d023311	Hap001	AA	233	
		Hap002	CC	65	Favorable
	Zm00001d023373	Hap001	AA	265	
		Hap002	CC	31	Favorable
RA	Zm00001d007563	Hap001	AA	259	
		Hap002	AA	41	Favorable
	Zm00001d039231	Hap001	TT	261	
		Hap002	GG	38	Favorable
	Zm00001d047812	Hap001	TT	238	
		Hap002	CC	49	Favorable
RDW	Zm00001d019169	Hap001	CC GG GG CC TT TT	243	
		Hap002	TT AA TT AA GG AA	49	Favorable
	Zm00001d019172	Hap001	TT	268	
		Hap002	AA	28	Favorable
	Zm00001d019173	Hap001	CC CC AA TT	268	
		Hap002	GG GG GG CC	28	Favorable
	Zm00001d019182	Hap001	GG	253	
		Hap002	AA	46	Favorable
	Zm00001d019184	Hap001	GG	251	
		Hap002	AA	46	Favorable
	Zm00001d019206	Hap001	AA	242	
		Hap002	CC	54	Favorable
	Zm00001d028225	Hap001	GG	213	Favorable
		Hap002	AA	87	
	Zm00001d047471	Hap001	CC	282	
		Hap002	TT	14	Favorable
	Zm00001d053375	Hap001	CC CC AA GG GG TT CC	264	Favorable
		Hap002	GG GG TT AA TT CC GG	33	
TNR	Zm00001d006496	Hap001	GG	259	Favorable
		Hap002	CC	41	
	Zm00001d038229	Hap001	TT GG	255	Favorable
		Hap002	GG AA	43	

The distribution of favorable haplotypes varied among maize subpopulations. Some favorable haplotypes were widely distributed across germplasm groups, whereas others exhibited distinct population-specific patterns (Fig. 6). Notably, the favorable haplotype of *Zm00001d038229*, which was associated with increased TNR, occurred at a higher frequency in tropical and admixed germplasms than in temperate germplasm.

**Fig. 7:**
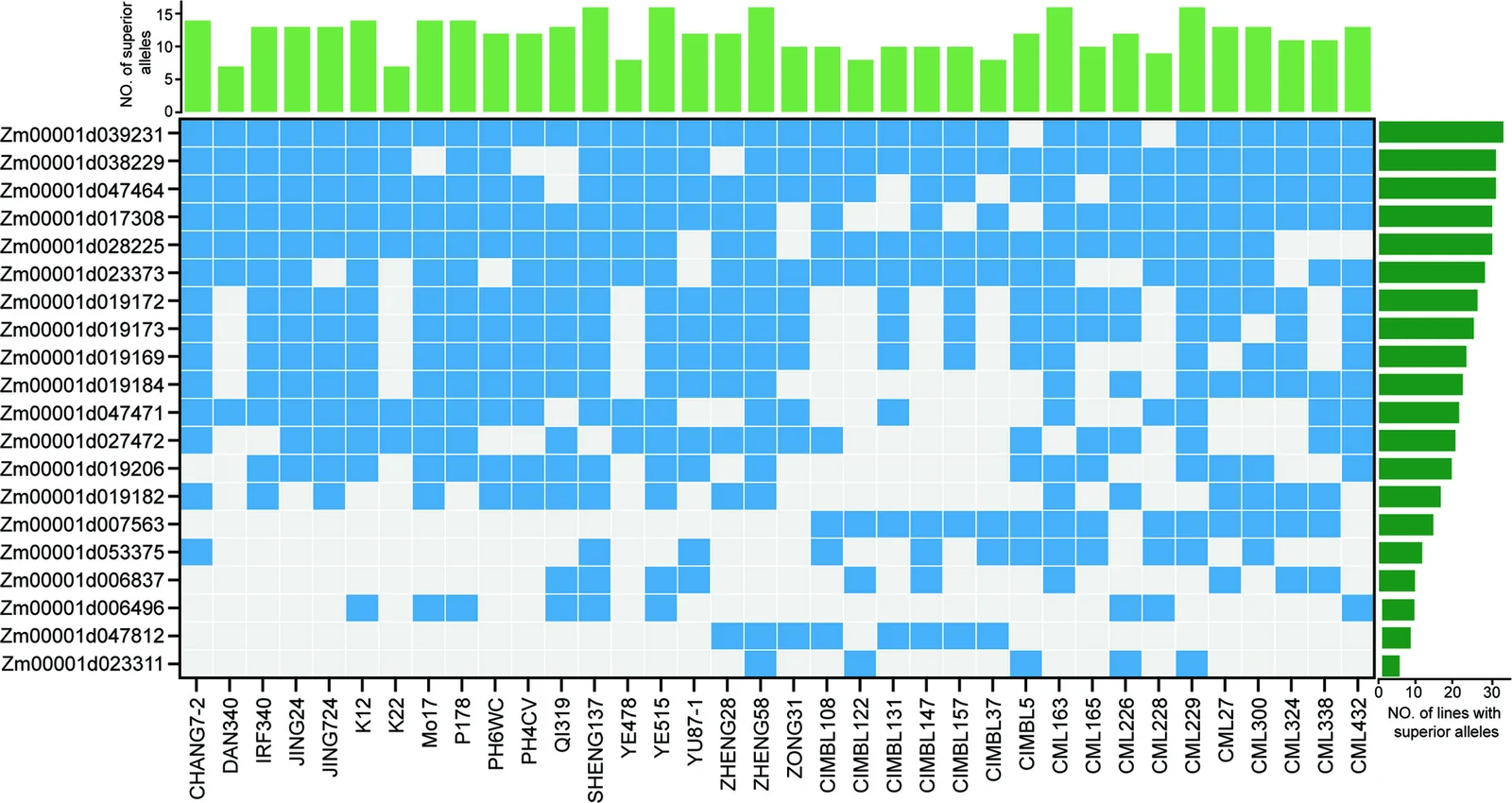
Distribution of favorable haplotypes in representative maize elite inbred lines. Blue cells indicate the presence of favorable haplotypes, whereas gray cells indicate their absence. Rows represent candidate genes and columns represent representative maize elite inbred lines. The bar chart above indicates the number of favorable haplotypes carried in each inbred line, and the bar chart on the right shows the number of inbred lines carrying the favorable haplotype for each gene. Favorable haplotypes were defined according to the haplotype analysis shown in Fig. 5

The distribution of favorable haplotypes was further evaluated in 36 representative maize inbred lines, including 16 widely used Chinese elite inbred lines and 20 tropical lines from CIMMYT. Substantial variation was observed in the number of favorable haplotypes carried by individual lines (Fig. 7). For example, Shen137, Ye515, Zheng58, CML163, and CML229 each carried 16 favorable alleles, whereas Dan340 and K22 carried only seven. Favorable haplotypes of *Zm00001d039231*, *Zm00001d038229*, *Zm00001d047464*, and *Zm00001d017308* were detected in more than 30 of the 36 lines, whereas those of *Zm00001d047812*, *Zm00001d023311*, and *Zm00001d006496* were detected in fewer than 10 lines. Notably, the favorable haplotype of *Zm00001d038229* was present in most CIMBL and CML lines but absent from several widely used temperate elite inbred lines, including Mo17, PH4VC, Qi319, and Zheng28. Overall, these results revealed substantial variation in the accumulation and distribution of favorable haplotypes among maize breeding materials.

### Functional characterization of *ZmGUX1* in maize nodal root development

**Fig. 8:**
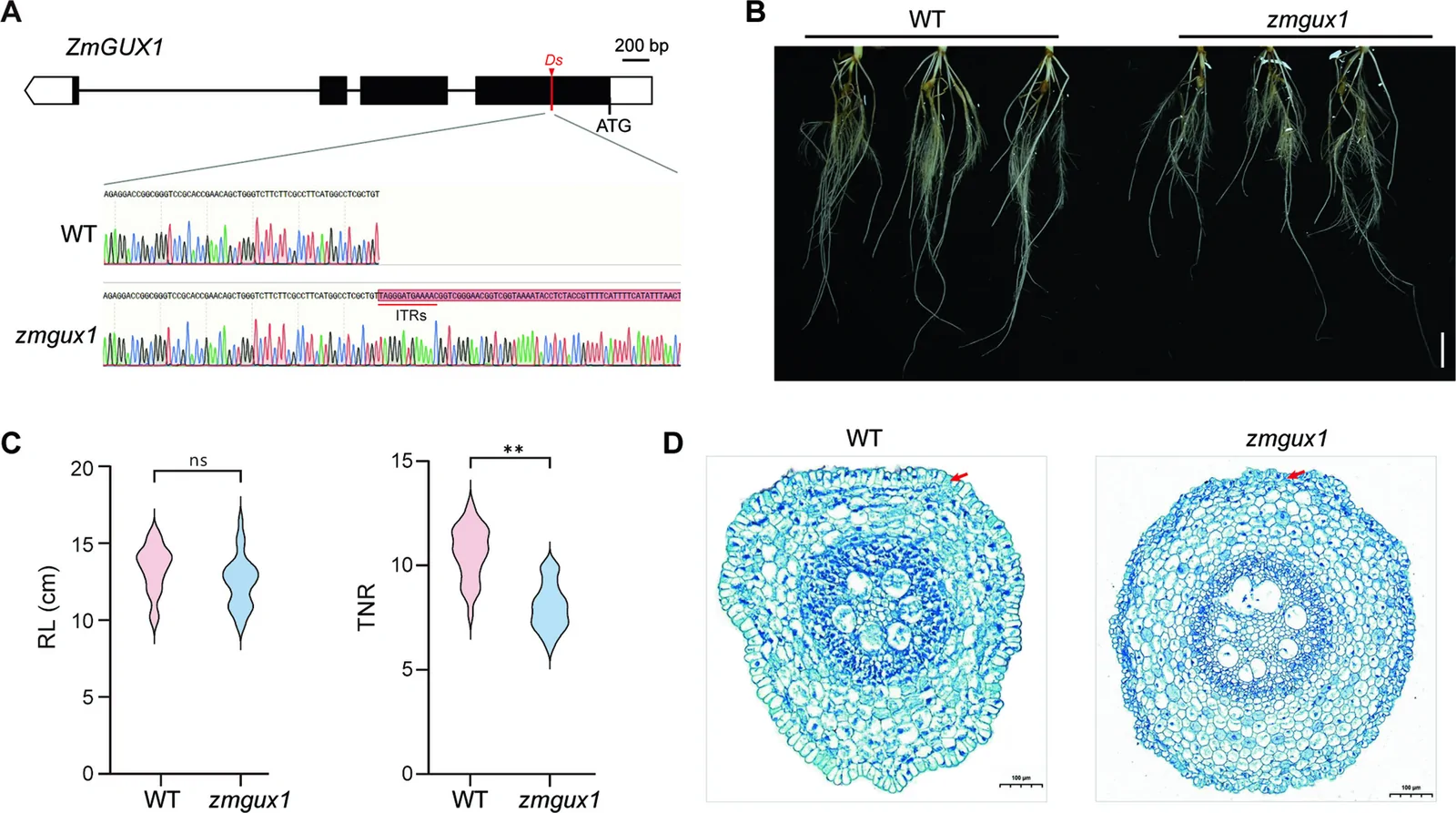
Functional characterization of *ZmGUX1* in maize nodal root development. **A** Identification of the *zmgux1 Ds* element insertion mutant. The *Ds* element was inserted 1,462 bp downstream of the translational start codon of *ZmGUX1*. PCR and sequencing confirmed the insertion site. **B** Seedling root phenotypes of WT and *zmgux1* mutant plants at the three-leaf stage. Bar = 2 cm. **C** Quantification of primary root length (RL) and total nodal roots number (TNR) at the three-leaf stage. Data are presented as mean ± SD. Asterisks indicate significant differences between WT and mutant plants (**P < 0.01).** D** Transverse sections of mature nodal roots from WT and *zmgux1* plants at the V6-stage. Arrows indicate representative epidermal cells. Scale bars = 100 μm

Among the candidate genes identified by GWAS, *Zm00001d038229* (*ZmGUX1*), which encodes a putative glycosyltransferase 8 protein, was selected for further characterization because its favorable haplotype was significantly associated with increased TNR. A *Ds* element insertion mutant was obtained from the *Ac/Ds* transposon library [30]. The mutant carried a transposon insertion 1,462 bp downstream of the translational start site of *ZmGUX1*, within the coding region of the gene (Fig. 8A).

Phenotypic analysis showed no significant difference in primary root length (RL) between WT and *zmgux1* seedlings. In contrast, TNR was significantly lower in the mutants than in WT plants (*P* < 0.01) (Fig. 8B, C), in agreement with the association between *ZmGUX1* and TNR identified by the GWAS and haplotype analyses. Anatomical examination of mature nodal roots revealed differences in tissue organization between the two genotypes. Compared with WT roots, *zmgux1* roots exhibited irregular arrangement of cell in the outer tissue layers and less organized cellular structure (Fig. 8D). Together, these results show that the *ZmGUX1* insertion mutation is associated with reduced nodal root number and altered root tissue organization, supporting the involvement of *ZmGUX1* in maize nodal root development.

## Discussion

### Joint analysis of stalk and root traits provides an integrated view of maize lodging resistance

Lodging resistance is a complex quantitative trait that contributes to maize yield stability under variable environmental conditions. Both stalk mechanical strength and root anchorage influence plant stability, and weakness in either component may increase lodging risk. Previous studies have identified numerous loci associated with stalk-related traits, including RPR, SBS, and SD, as well as loci associated with root architectural traits such as TNR, RA, and RD [13, 22, 24]. However, most previous studies examined stalk- and root-related traits separately, leaving their combined genetic architecture less well characterized.

In the present study, five stalk mechanical traits and five root architectural traits were evaluated simultaneously in a diverse panel of 300 maize inbred lines across three environments. All traits showed relatively high broad-sense heritability, ranging from 72.2% to 94.7%, indicating that genetic variation accounted for a substantial proportion of the observed phenotypic variation. Significant correlations were detected within the stalk- and root-related trait groups, as well as between several stalk and root traits. The correlations suggest that variation in stalk mechanical properties and root architecture may jointly influence plant structural stability. This interpretation is consistent with previous reports showing that both stalk strength and root anchorage are relevant to maize lodging resistance [31, 32]. Nevertheless, the traits measured in this study represent component phenotypes related to lodging resistance rather than direct measurements of field lodging. Stalk mechanical traits mainly reflect the capacity of the stem to resist bending, crushing, or breakage, whereas root architectural traits are related to anchorage and root-soil interactions. Actual field lodging is additionally influenced by wind speed, rainfall, soil moisture, planting density, developmental stage, and crop management. Therefore, the identified associations should be interpreted as loci related to structural traits that may contribute to lodging resistance. Their effects on stalk lodging and root lodging under natural field conditions require further evaluation across diverse environments and management systems.

The GWAS results further illustrated the quantitative nature of the investigated traits. The number of significant SNPs differed markedly among traits and statistical models. For several traits, the MLM detected fewer significant associations than the GLM or FarmCPU. This difference may reflect the conservative control of population structure and kinship in the MLM, which can reduce power for detecting loci with relatively small effects. In contrast, the GLM may provide greater detection power but can be more sensitive to population structure, whereas the FarmCPU attempts to balance statistical power and false-positive control through iterative fixed- and random-effect modeling.

Across the ten traits, 1,175 significant SNPs were identified, of which 458 were detected by at least two GWAS models. Repeated detection across models increases confidence in these association signals, although independent validation remains necessary. Most detected loci explained small-to-moderate proportions of phenotypic variation, which is consistent with the polygenic inheritance of lodging-related traits reported in previous studies [7, 14].

No obvious overlap was observed between the loci identified here and previously reported QTLs or GWAS signals. This limited concordance may result from differences in genetic background, environmental conditions, phenotyping methods, marker density, linkage disequilibrium patterns, and statistical models. In addition, the present study jointly evaluated stalk mechanical and root architectural traits, whereas many earlier studies focused on one trait category. Thus, the current findings complement previous research by providing an integrated assessment of genetic variation associated with both stalk and root components of maize lodging resistance.

### Candidate genes suggest possible roles of cell wall metabolism and tissue development in lodging-related traits

Several candidate genes identified in this study were annotated as being involved in cell wall polysaccharide metabolism, glycosylation, or tissue development, suggesting that variation in these processes may contribute to stalk mechanical and root architectural traits. However, these functional interpretations are mainly based on gene annotation, expression patterns, and association results and therefore require further experimental validation.

Plant mechanical strength is closely related to the composition and organization of the cell wall. In grasses, cellulose, lignin, and glucuronoarabinoxylans (GAXs) form major structural components of supporting tissues [33]. Consistent with this, *Zm00001d027472* encodes a putative callose synthase, a key β−1,3-glucan component of plant cell walls. Maize callose synthase genes have been implicated in vegetative development and vascular bundle formation [34]. *Zm00001d040684* encodes a putative pectin acetylesterase family protein, and *Zm00001d039231* is predicted to participate in polysaccharide biosynthesis and vascular development. Recent studies have shown that vascular tissue organization contributes substantially to stalk support and root anchorage, indicating that variation in structural tissue formation may influence lodging resistance through changes in mechanical strength [35]. Arabidopsis GUX proteins function in glucuronoxylan modification, which is important for secondary wall organization [36, 37]. The association of *ZmGUX1* with TNR, together with its root expression pattern and mutant phenotype, supports its possible involvement in nodal root development. Overall, the candidate genes identified here point to cell wall metabolism and tissue development as plausible biological processes associated with variation in maize lodging-related traits. Their specific functions and underlying mechanisms remain to be determined through further genetic and biochemical analyses.

### Favorable haplotypes represent candidate resources for maize improvement

Significant phenotypic differences among haplotypes were detected for 20 of the 26 candidate genes. These results indicate associations between natural allelic variation at these loci and lodging-related traits. However, haplotype-phenotype associations may also be influenced by linkage disequilibrium with nearby causal variants. Therefore, the identified favorable haplotypes should be considered candidate breeding resources that require validation in independent populations and genetic backgrounds.

The distribution of favorable haplotypes varied among maize germplasms groups. Evaluation of 36 representative inbred lines revealed substantial differences in the number of favorable haplotypes carried by individual lines. Several favorable haplotypes were common across the surveyed materials, whereas others occurred at relatively low frequencies. Similar uneven distributions of alleles associated with stalk lodging resistance have been reported in elite maize germplasm [38]. The absence of some favorable haplotypes from widely used temperate inbred lines suggests that additional allelic variation remains available for potential use in breeding.

Several favorable haplotypes were more frequent in CIMMYT-derived tropical lines than in the temperate materials examined in this study. Tropical maize germplasm contains broad genetic diversity and has long been considered a useful source of allelic variation for maize improvement [38, 39]. At the same time, domestication and modern breeding have reduced genetic diversity in some elite germplasm pools through bottlenecks and selection [40, 41]. The observed distribution patterns are compatible with the possibility that tropical germplasm contains useful variation for lodging-related traits. However, the present survey included only 36 representative lines, and broader germplasm evaluation is needed before general conclusions can be drawn about population-wide enrichment.

Following validation across diverse genetic backgrounds and field environments, the identified haplotypes may serve as candidate markers for marker-assisted selection or as features incorporated into genomic prediction models. Combining favorable haplotypes associated with stalk mechanical strength and root architectural traits may provide a strategy for improving multiple structural components of lodging resistance. However, possible trade-offs with flowering time, plant height, biomass allocation, and yield should also be evaluated before these haplotypes are used in breeding programs.

### *ZmGUX1* is associated with nodal root development in maize

Among the candidate genes identified by GWAS, *ZmGUX1* was selected for further characterization because it was significantly associated with TNR and showed significant haplotype effects. *ZmGUX1* encodes a putative glycosyltransferase 8 family protein. Studies in Arabidopsis have showed that GUX proteins act as xylan glucuronosyltransferases and contribute to glucuronoxylan substitution patterns in secondary cell walls [36, 37].

Xylan is a major hemicellulosic component of secondary cell walls and contributes to wall organization and tissue mechanical properties. Genes belonging to the GT8, GT43, and GT47 families have been associated with xylan biosynthesis and secondary wall development in model plant species [42–45]. Mutations in some xylan biosynthetic genes can alter secondary wall thickening and xylem morphology. In addition, *GUX1* and *GUX2* produce different glucuronoxylan substitution patterns that may affect interactions among xylan, cellulose, and lignin [46, 47].

In the present study, the *zmgux1* insertion mutant had fewer nodal roots and showed altered cellular organization in mature nodal roots, whereas RL did not differ significantly from that of WT plants. These observations support an involvement of *ZmGUX1* in nodal root development and suggest that its effect may be more closely related to post-embryonic root formation or tissue organization than to general primary root elongation.

The mutant phenotype was directionally consistent with the GWAS and haplotype associations for TNR. Nevertheless, several limitations should be considered. The study examined one insertion mutant, and the effect of the insertion on *ZmGUX1* transcript or protein abundance was not determined. In addition, complementation and analysis of independent alleles were not performed. Thus, although the results support *ZmGUX1* as a candidate gene involved in nodal root development, further genetic evidence is required to establish causality.

Nodal roots contribute to maize root system architecture and may influence plant anchorage. However, root anchorage strength and actual field lodging were not measured in the mutant. Therefore, the present data do not directly demonstrate that *ZmGUX1* controls lodging resistance. Future studies should evaluate independent mutant alleles, complementation lines, cell wall composition, root biomechanical properties, and lodging performance under field conditions. Such analyses will clarify whether the effect of *ZmGUX1* on nodal root development translates into measurable differences in root anchorage or lodging resistance.

## Conclusions

This study jointly investigated five stalk mechanical traits and five root architectural traits in a diverse maize association panel, providing an integrated analysis of the genetic basis of lodging-related traits. GWAS identified 1,175 significant SNPs and 26 candidate genes, several of which are predicted to be involved in cell wall metabolism and tissue development. Haplotype analysis identified favorable haplotypes associated with lodging-related traits and revealed substantial variation in their distribution among maize germplasm. In addition, functional characterization of *ZmGUX1* supported its involvement in maize nodal root development. Together, these findings expand our understanding of the genetic architecture underlying maize lodging-related traits and provide candidate genes and haplotypes for further functional validation. Following validation in diverse genetic backgrounds and field environments, these genetic resources may facilitate marker-assisted selection and genomic breeding for improved maize structural stability.

## Supplementary Information

Supplementary Material 1: Figure S1. Distribution characteristics of raw SNP datasets before quality control. Histograms showing the distributions of individual missingness, SNP missingness, MAF, and Hardy–Weinberg equilibrium (HWE) *P* values in the raw SNP dataset generated from resequencing of 300 maize inbred lines. Figure S2. Distribution characteristics of high-quality SNP datasets after quality control. Histograms showing the distributions of individual missingness, SNP missingness, MAF, and HWE *P* values after SNP quality filtering. SNPs with a MAF < 0.05 and a missing rate > 0.01 were removed. Figure S3. Genome-wide distribution of high-quality SNPs across the maize genome. Heatmap showing the density distribution of 1,213,985 high-quality SNPs across the ten maize chromosomes using a 1-Mb sliding window. Warmer colors indicate higher SNP densities.

Supplementary Material 2: Table S1. Summary of significant SNPs identified for lodging-related traits by different GWAS models. Table S2. Significant SNPs associated with lodging-related traits identified by GWAS.

## Data Availability

The SNP data generated and analyzed during this study are available under accession number GVM001453 at https://ngdc.cncb.ac.cn/gvm.
