# Supplementary material for: Genome-wide association study identifies candidate genes and favorable haplotypes for maize lodging-related traits
Source: BMC Plant Biol. 2026 Aug 14;26:1932. doi: 10.1186/s12870-026-09725-x (PMC13753391; doi:10.1186/s12870-026-09725-x)
Supplement: Supplementary file 1 [file 12870_2026_9725_MOESM1_ESM.docx]

**Figure S1. Distribution characteristics of raw SNP datasets before quality control.** Histograms showing the distributions of individual missingness, SNP missingness, MAF, and Hardy–Weinberg equilibrium (HWE) *P* values in the raw SNP dataset generated from resequencing of 300 maize inbred lines.

**Figure S2. Distribution characteristics of high-quality SNP datasets after quality control.** Histograms showing the distributions of individual missingness, SNP missingness, MAF, and HWE *P* values after SNP quality filtering. SNPs with a MAF < 0.05 and a missing rate > 0.01 were removed.

**Figure S3. Genome-wide distribution of high-quality SNPs across the maize genome.** Heatmap showing the density distribution of 1,213,985 high-quality SNPs across the ten maize chromosomes using a 1-Mb sliding window. Warmer colors indicate higher SNP densities.
